# Multiple Linear Regression Analysis of lncRNA–Disease Association Prediction Based on Clinical Prognosis Data

**DOI:** 10.1155/2018/3823082

**Published:** 2018-12-11

**Authors:** Bo Wang, Jing Zhang

**Affiliations:** ^1^College of Computer Science and Technology, Harbin Engineering University, Harbin 150001, China; ^2^College of Computer and Control Engineering, Qiqihar University, Qiqihar 161006, China; ^3^Department of Computer Science and Technology, Institute of Technology, Shantou University, Shantou 515063, China; ^4^School of Information Science and Engineering, University of Jinan, Jinan 250022, China

## Abstract

Long noncoding RNAs (lncRNAs) have an important role in various life processes of the body, especially cancer. The analysis of disease prognosis is ignored in current prediction on lncRNA–disease associations. In this study, a multiple linear regression model was constructed for lncRNA–disease association prediction based on clinical prognosis data (MlrLDAcp), which integrated the cancer data of clinical prognosis and the expression quantity of lncRNA transcript. MlrLDAcp could realize not only cancer survival prediction but also lncRNA–disease association prediction. Ultimately, 60 lncRNAs most closely related to prostate cancer survival were selected from 481 alternative lncRNAs. Then, the multiple linear regression relationship between the prognosis survival of 176 patients with prostate cancer and 60 lncRNAs was also given. Compared with previous studies, MlrLDAcp had a predominant survival predictive ability and could effectively predict lncRNA–disease associations. MlrLDAcp had an area under the curve (AUC) value of 0.875 for survival prediction and an AUC value of 0.872 for lncRNA–disease association prediction. It could be an effective biological method for biomedical research.

## 1. Introduction

Long noncoding RNAs (lncRNAs) are noncoding RNA molecules, including miRNAs [1], lncRNAs [2], tRNAs [3], piRNAs [4], and more than 200 nucleotides. They were initially thought to be nonfunctional RNA fragments and the only by-product of massive transcription [5–8]. A large number of recent studies have shown that lncRNAs have abundant biological functions, including the silencing of X chromosome [9] and activation and interference of transcription [10–12]. At the same time, the abnormal expression of lncRNAs leads to various diseases [13–15]. Therefore the investigation on lncRNA–disease associations is of great significance at the molecular level to radically cure the disease.

Many computational methods have been applied to human lncRNA–disease association prediction in recent years. These methods have two prominent features: machine-learning-based feature and network-based feature.

The machine-learning-based feature of lncRNA–disease association prediction is to establish a learning model in the training dataset and then to perform tests in the test dataset using this learning model. For instance, Zhao et al. [16] developed a learning model based on the Bayesian classifier for lncRNA–disease association prediction. The key issue was that the learning model regarded unknown lncRNA–disease associations as negative sets, restricting the performance of the learning model. In fact, the negative sets for lncRNA–disease association prediction were difficult to achieve. To avoid this problem, Chen et al. [17] put forward the method of LRLSLDA to predict lncRNA–disease associations. It was based only on positive sets, not on negative sets. It adopted the strategy of Laplacian regularized least squares, was a semisupervised learning model, and needed selected optimal parameters to obtain optimal prediction results. LRLSLDA had two limitations: (a) they were under the assumption that functionally similar lncRNAs were related to similar diseases and (b) they were restricted to selecting optimal parameters. Moreover, Chen et al. [18] developed another method, KATZLDA, which incorporated known lncRNA–disease associations, lncRNA expression profiles, lncRNA functional similarity, disease semantic similarity, and Gaussian interaction profile kernel similarity. KATZLDA could adapt to new diseases and lncRNAs without any known associations. However, KATZLDA still was built on lncRNAs with more known associated diseases or/and miRNA interaction partners.

The network-based feature of lncRNAs–disease association prediction is to establish a learning network of lncRNA–disease associations using known associations. For instance, Yang et al. [19] constructed the bipartite network about lncRNA–disease associations and predicted these associations by the method of transmission in the network, which was the first prediction method based on the network model. A coding–noncoding gene–disease bipartite network was constructed to improve the prediction results in which better prediction results were obtained. However, the approach did not take into account the interaction between lncRNAs and coding genes, and the forecast range was limited. The results were relatively general. Sun et al. [20] proposed a new method based on the network model: RWRlncD. It was used to construct the functional similarity network of lncRNAs using the known lncRNA–disease association network and the similarity network. Subsequently, the reactivated random walk was performed in the functional similarity network of lncRNAs to predict the potential lncRNA–disease associations. However, the edge of the test set was used to calculate the functional similarity of lncRNAs before cross validation, which overestimated the verification results. Zhou et al. [21] presented a novel method (named RWRHLD) to distinguish candidate lncRNA–disease associations using the hybrid network and then performed the random walk algorithm on this hybrid network. RWRHLD was used only to predict lncRNAs in known lncRNA–miRNA associations, where an incomplete coverage of lncRNAs cross talk network and lncRNA–disease association network might lead to inaccurate prediction results. Chen et al. [22] improved the traditional random walk with restart and proposed the method of improved random walk with restart for lncRNA–disease association prediction (IRWRLDA). But the existing problem of IRWRLDA was how to obtain integrated lncRNA similarity based on lncRNA functional similarity and lncRNA Gaussian interaction profile kernel similarity. Chen et al. [23] developed two novel LNCSIMs and proposed a new method LRLSLDA–LNCSIM that could improve the predictive ability of LRLSLDA. LRLSLDA–LNCSIM still had the limit that a semantic contribution decay factor was not well solved. Yu et al. [24] employed multidimensional heterogeneous data to construct an lncRNA function similarity network, employed the disease ontology to construct a disease network, and then proposed the BRWLDA to predict lncRNA–disease associations. Although the prediction performance was improved by BRWLDA, the defect of random walk algorithm still existed. Chen et al. [25] developed a method of HGLDA by integrating miRNA–disease associations and lncRNA–miRNA interactions. However, HGLDA could not be used in the lncRNAs without any known miRNA interaction partners. Ganegoda et al. [26] developed the computational model of KRWRH network, which was a heterogeneous network formed by integrating a disease–disease similarity network, lincRNA–lincRNA similarity network, and known lincRNA–disease association network.

The reviews of Chen et al. [27] and the aforementioned discussions showed that few references were made to the combination of clinical prognosis data with lncRNA–disease association in the existing studies on lncRNA–disease association prediction. In the present study, the analysis of disease prognosis was ignored, and the existing prediction model was limited to a single lncRNA prediction. The prognosis information of a disease associated with lncRNAs was rarely involved (such as the survival time of patients, current state of disease, and family history of genetic diseases). In fact, an analysis related to the prognosis of lncRNA–disease association has more realistic meaning and value.

To overcome the aforementioned issues, a multiple linear regression model for lncRNA–disease association prediction based on clinical prognosis data (MlrLDAcp) was constructed to predict the potential associations between lncRNAs and diseases. At the same time, the survival time of patients with prostate cancer was also predicted in MlrLDAcp. The concepts of predictive correlation factor Θ, decay coefficient *ξ*, Γ operation, and Γ correction were proposed in this study to construct the multiple linear regression model. An algorithm for developing the multiple linear regression model was designed, in which 481 lncRNA transcripts with *P* values less than 0.001 were cut back to 60 most closely related to the survival time of patients with prostate cancer. Finally, the potential multiple linear regression relationship between the prognosis survival time of 176 patients with prostate cancer and 60 lncRNAs was proposed. MlrLDAcp could realize not only cancer survival prediction but also lncRNA–disease association prediction. Compared with previous findings, MlrLDAcp had a predominant survival predictive ability and could effectively predict lncRNA–disease associations.

## 2. Materials and Methods

### 2.1. LncRNA Expression Data

The lncRNA expression data of prostate cancer was obtained from the lncRNAtor database (http://lncrnator.ewha.ac.kr). A total of 44 normal samples and 176 prostate cancer samples were obtained, and the prostate cancer samples were denoted in an ascending order according to sample ID (denoted by *T*_1_, *T*_2_, ⋯, *T*_176_). The expression level of each lncRNA transcript in normal and prostate cancer samples was calculated. The differential expressions between normal and prostate cancer samples were calculated using the aforementioned expression quantities. A total of 481 lncRNA transcripts with a significant difference were obtained (denoted by *G*_1_, *G*_2_, ⋯, *G*_481_ using a* P* value in the ascending order) by selecting a* P* value less than 0.001, and the 481 transcript expression quantities on *T*_*i*_ were denoted by *G*_*i*~1_, *G*_*i*~2_, ⋯, *G*_*i*~481_(1 ≤ *i* ≤ 176). The lncRNA expression training data matrix (denoted by $Led=\left[\begin{array}{cccc} G_{1\sim 1} & G_{1\sim 2} & \cdots & G_{1\sim 481}\\ G_{2\sim 1} & G_{2\sim 2} & \cdots & G_{2\sim 481}\\ \vdots & \vdots & \vdots & \vdots \\ G_{176\sim 1} & G_{176\sim 2} & \cdots & G_{176\sim 481} \end{array}\right]$) was constructed based on the aforementioned data.

### 2.2. Clinical Prognosis Data of Patients with Prostate Cancer

The clinical prognosis data of 176 prostate cancer samples in Section 2.1 were obtained from the TCGA database (https://cancergenome.nih.gov). Each prostate cancer sample contained the clinical prognostic data of 60 samples. The data were filtered to keep the patient ID (submitter_id), survival state (vital_status, the survival state of *T*_*i*_ denoted by *Vs*_*i*_), time of death of patients (days_to_death, the death time of *T*_*i*_ denoted by *Dd*_*i*_), and final contact time (days_to_last_follow_up, final contact time of *T*_*i*_ denoted by *Dl*_*i*_). Hence, the survival time training matrix $\omega =\left[\begin{array}{cc} Dd_{1} & Dl_{1}\\ Dd_{2} & Dl_{2}\\ \vdots & \vdots \\ Dd_{176} & Dl_{176} \end{array}\right]$ was obtained. If the patient was in a state of death, he had only the time of death but no final contact time, and the final contact time was recorded as 0. On the contrary, if the patient was alive, he had only the final contact time but no death time, and the death time was recorded as 0. Therefore, the survival distribution coefficient matrix $\Omega =\left[\begin{array}{c} \alpha \\ \beta \end{array}\right]$ (if *Vs*_*i*_ = *dead* then *α* = 1, *β* = 0; else *α* = 0, *β* = 1) was constructed. Finally, the survival analysis matrix $La=\omega \times \Omega =\left[\begin{array}{cc} Dd_{1} & Dl_{1}\\ Dd_{2} & Dl_{2}\\ \vdots & \vdots \\ Dd_{176} & Dl_{176} \end{array}\right]\times \left[\begin{array}{c} \alpha \\ \beta \end{array}\right]$ was obtained.

### 2.3. Abstracting the Issue

This study on lncRNA–disease associations was conducted from the following two aspects:

(a) A part of lncRNAs in the 481 lncRNAs, which were most closely related to prostate cancer, was screened out through the analysis of prognosis survival. Hence, a subset of *Led* (denoted by *Led*^*sub*^, *Led*^sub^ ⊂ *Led*) was obtained, and set Δ = 〈*Led*^*sub*^〉 (〈*Led*^*sub*^〉 was the number of elements in *Led*^*sub*^, 1 ≤ Δ < 481). *Led*^*sub*^ contained Δ lncRNAs (denoted by *g*_1_, *g*_2_, ⋯, *g*_Δ_). The expression quantity of *g*_*i*_ on *T*_*i*_ was denoted by *g*_*i*~1_, *g*_*i*~2_, ⋯, *g*_*i*~Δ_. Therefore, $Led^{sub}=\left[\begin{array}{cccc} g_{1\sim 1} & g_{1\sim 2} & \cdots & g_{1\sim \Delta }\\ g_{2\sim 1} & g_{2\sim 2} & \cdots & g_{2\sim \Delta }\\ \vdots & \vdots & \vdots & \vdots \\ g_{176\sim 1} & g_{176\sim 2} & \cdots & g_{176\sim \Delta } \end{array}\right]$.

(b) The potential relationship between *Led*^*sub*^ and *La* was given using multiple statistical methods, and finally the prognosis prediction of lncRNA–disease associations was realized using *Led*^*sub*^ predicting *La*.

**Definition 1 (predictive correlation factor Θ).**

Θ was defined as $\Theta =\left[\begin{array}{c} \Theta_{1}\\ \Theta_{2}\\ \vdots \\ \Theta_{\Delta } \end{array}\right]$, where Θ_*i*_ corresponded to *G*_*i*_ in *Led*^*sub*^. The value of predictive correlation factor Θ_*i*_ was the coefficient of multiple linear regression *La*. *La* is shown in (1). For the prognosis prediction of lncRNA–disease associations, the formal definition was as follows. Two tasks needed to be completed while establishing *La*: (1) to calculate *Led*^*sub*^ and (2) to calculate Θ.

(1) La=Ledsub×Θ=g1~1g1~2⋯g1~Δg2~1g2~2⋯g2~Δ⋮⋮⋮⋮g176~1g176~2⋯g176~Δ×Θ1Θ2⋮ΘΔ=Dd1Dl1Dd2Dl2⋮⋮Dd176Dl176×αβ

### 2.4. Γ Operation

**Definition 2 (line class vector *X*).**

*X* was a one-dimensional vector with 481 rows and 1 column (*X* = [*X*_1_, *X*_2_, ⋯, *X*_481_]). *X*_*i*_ corresponded to *G*_*i*_, and that *X*_*i*_ was equal to 1 or 0. When *X*_*i*_ was equal to 1, *G*_*i*_ corresponding to *X*_*i*_ was selected to *Led*^*sub*^. Otherwise, *G*_*i*_ corresponding to *X*_*i*_ was not selected to *Led*^*sub*^. *C*(*X*) was the number of *X* components (i.e., 481). *C*(*X*)^=1^ was the number of *X* components, the value of which was 1.

**Definition 3 (decay coefficient *ξ*).**

*ξ* denoted the decay duration in Γ operation. *ξ*(*d*), which was the *ξ* value of the *dth* iteration in Γ operation, is shown in (2).

(2) ξd=0.1d∈Z,10≤d≤De2×D−d/D+1−e1/2×D−d/D+1e2×D−d/D+1+e1/2×D−d/D+1d∈Z,1≤d≤9

In (2), *D* is the maximal iterations of Γ operation and *d* is the current iterations of Γ operation. When *d* takes the minimal value 1, *ξ* is close to 1. When *d* takes the maximal value *D*, *ξ* is 0.1. When *d* takes the value between 1 and *D*, *ξ* is the reduction value between 1 and 0.1. The greater *ξ* is, the greater the decay efficiency is.

**Definition 4 (Γ operation).**

**Figure 1:**
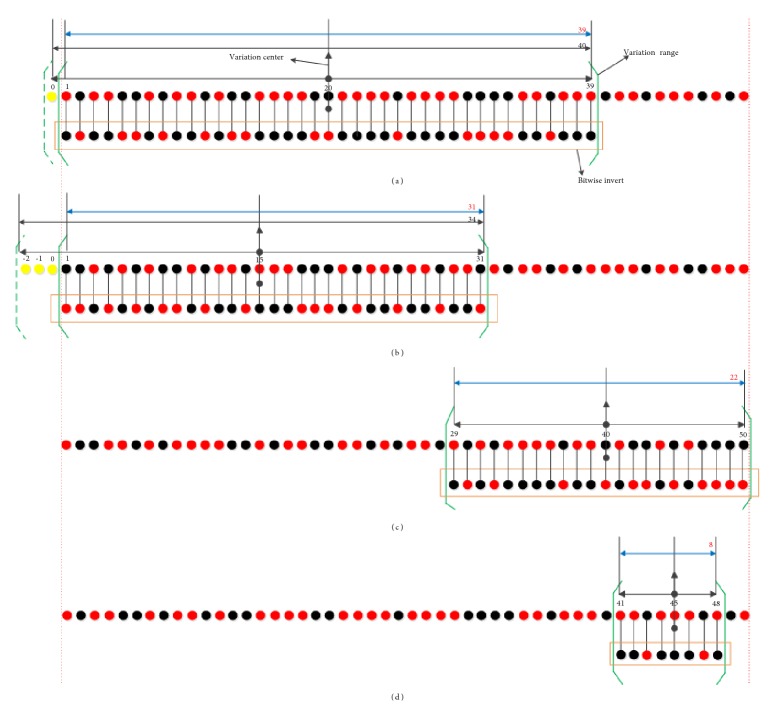
Schematic of Γ operation. The black circles represent 0. The red circles represent 1. The yellow circles represent overflow value. The green curly bracket area represents bitwise invert. To briefly explain the principle, the principle parameters were set as follows: *C*(*X*) = 50, *D* = 10. The number of iterations of (a) was 2. The number of iterations of (b) was 4. The number of iterations of (c) was 7. The number of iterations of (d) was 9. (a) Calculated that *Center*(*X*^1^) = 20, *Range*(*X*^1^) = [1,39], the length of Γ operation was 39, and the left side overflowed a value. (b) Calculated that *Center*(*X*^4^) = 15, *Range*(*X*^4^) = [1,31], the length of Γ operation was 31, and the left side overflowed three values. (c) Calculated that *Center*(*X*^6^) = 40, *Range*(*X*^6^) = [29,50], and the length of Γ operation was 22. (d) Calculated that *Center*(*X*^9^) = 45, *Range*(*X*^9^) = [41,48], and the length of Γ operation was 8.

Γ operation was divided into three stages. (a) The variation center *Center*(*X*^*d*^) of the *dth* iteration on *X* was calculated according to (3). (b) The variation range *Range*(*X*^*d*^) of the *dth* iteration on *X* was calculated according to (4). (c) Bitwise inversion was performed within the variation range (*Range*(*X*^*d*^)∩[1, *C*(*X*)]).  Figure 1 shows the schematic of Γ operation.

(3) CenterXd=CX2d=1CX2+CXD×−1d−1×d22≤d≤D

(4) RangeXd=CenterXd−ξd×CX2,CenterXd+ξd×CX2−1

In (3) and (4), *D* is the maximal iterations of Γ operation and *d* is the current iterations of Γ operation.

**Definition 5 (Γ correction).**

The components (their value was 1) in *X*^*d*^ remained valid only within the top *θ* period in Γ operation, and the rest were set to 0.

### 2.5. Stepwise

*Stepwise*(*X*^*d*^) was performed *Stepwise* on *X*^*d*^. {*X*_+_^*d*^} was the set of *X*_*j*_^*d*^ = 1. The insignificant component *X*_*j*_^*d*^ in *X*^*d*^ went from 1 to 0 by *Stepwise*(*X*^*d*^). After executing* Stepwise*, {*X*_+_^*d*^} was changed to {−*X*_+_^*d*^}. *Stepwise*(*X*^*d*^) was the process of further subtracting and retaining the most important components. The execution of* Stepwise* went through the following six steps. (A temporary container {*X*^*temp*^} with an initial value of empty and two marker variables *flag*_+_ and *flag*_−_ were defined. The initial values of both marker variables were 0.)

**Step 1 .**

A component *X*_*j*_^*d*^ of significant effect on *La* was added to {*X*^*temp*^}.

**Step 2 .**

Whether a new component was added to {*X*^*temp*^} was judged; if true, then both *flag*_+_ and *flag*_−_ were set to 0 and Step 1 was performed; otherwise, *flag*_+_ was set to 1 and Step 3 was performed.

**Step 3 .**

Whether *flag*_+_ = 1 and *flag*_−_ = 1 was judged; if true, then Step 6 was performed; otherwise, Step 4 was performed.

**Step 4 .**

A component *X*_*j*_^*d*^ of insignificant effect on *La* was removed from {*X*^*temp*^}.

**Step 5 .**

Whether a new component was removed from {*X*^*temp*^} was judged; if true, then both *flag*_+_ and *flag*_−_ were set to 0 and Step 4 was performed; otherwise, *flag*_−_ was set to 1 and Step 1 was performed.

**Step 6 .**

The components in {*X*_+_^*d*^}, which was not in {*X*^*temp*^}, were set to 0. The updated {*X*_+_^*d*^} was set to {−*X*_+_^*d*^}, then {−*X*_+_^*d*^} and *AIC*({−*X*_+_^*d*^}) were the output.

### 2.6. Algorithm of Computing *La*

**Figure 2:**
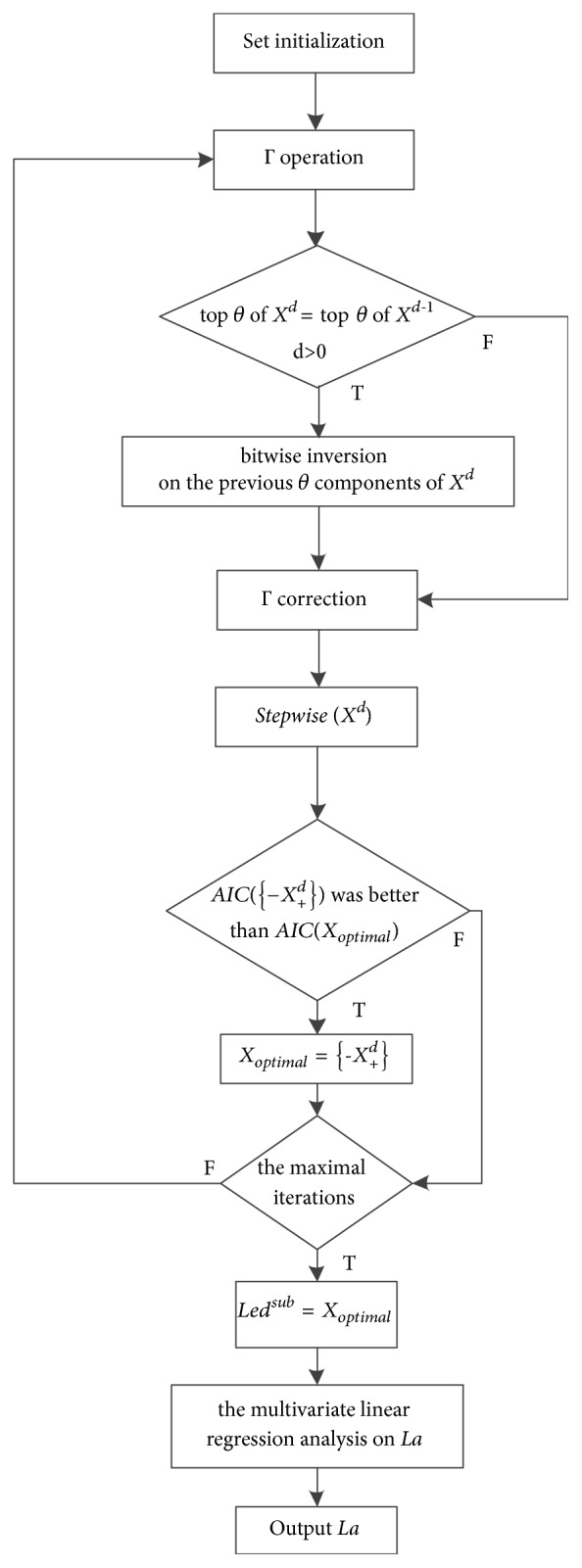
Algorithm flowchart of computing *La*.

The algorithm flow of computing *La* was as follows. (Figure 2 shows the algorithm flowchart of computing *La*.)

**Step 1 .**

Initialization was set. *D* = 10, *θ* = *C*(*X*^0^)^=1^, *C*(*X*) = 481, *X*_*optimal*_ = *X*^0^, and *AIC*(*X*_*optimal*_). *X*_*optimal*_ was the current best bulletin board. *X*_*i*_^0^, the *P* value of which was less than 10^−12^ in *X*, was set to 1, and the rest was set to 0.

**Step 2 .**

Γ operation was executed. If top *θ* components of *X*^*d*^ were the same as top *θ* components of *X*^*d*−1^ and *d* > 0, bitwise inversion was performed on the previous *θ* components of *X*^*d*^. After that, Γ correction was performed.

**Step 3 .**

*Stepwise*(*X*^*d*^) was executed. If *AIC*({−*X*_+_^*d*^}) was better than *AIC*(*X*_*optimal*_), then *X*_*optimal*_ = {−*X*_+_^*d*^}, and *AIC*(*X*_*optimal*_) was updated.

**Step 4 .**

Whether the maximal iterations were reached was judged; if true, then Step 5 was performed; otherwise, Step 2 was performed.

**Step 5 .**

*Led* ^*sub*^ was set to *X*_*optimal*_.

**Step 6 .**

The multivariate linear regression analysis on *La* was performed using *Led*^*sub*^. Θ was set to the multiple linear regression coefficients.

**Step 7 .**

*La* was the output.

## 3. Results and Discussion

### 3.1. *Center*(*X*^*d*^)

**Figure 3:**
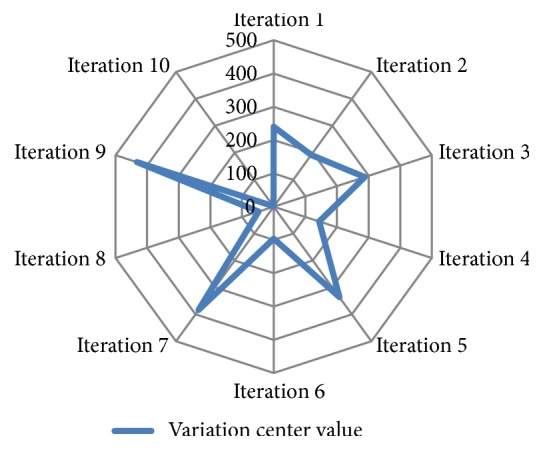
Distribution of the variation center on Γ operation among 10 iterations.

The calculation results of variation center *Center*(*X*^*d*^) in Γ operation are shown in Figure 3. The figure shows that the variation center value of Γ operation was evenly distributed in 10 iterations. Furthermore, the variation center value was scattered around each interval from 1 to 481. The variation center value had 5 points on each side of the center point (240). This ensured that the components of all 481 LncRNAs had an equal opportunity to perform variations. It was more beneficial to obtain the global optimal solution.

### 3.2. *ξ*(*d*)

**Figure 4:**
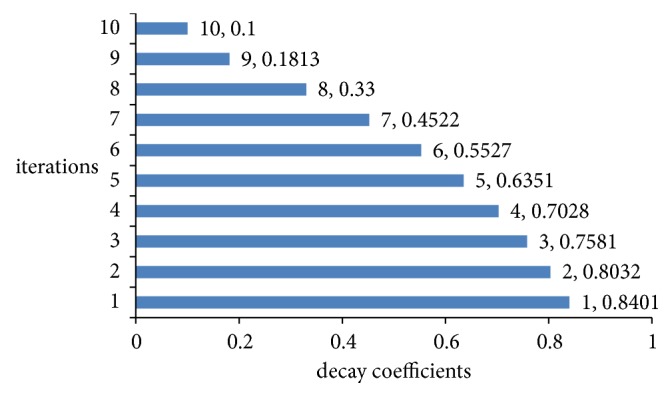
Distribution of the decay coefficients of the Γ operation among 10 iterations.

Figure 4 shows the calculation results of decay coefficient *ξ*. The variation operation proposed in this study aimed to enrich the diversity of sample space. Meanwhile, the variation operation should be dynamic rather than fixed. For the aforementioned issue, the decay coefficient *ξ* was proposed to control the strength of the variation operation. Figure 4 shows that the decay coefficient *ξ* decreased with the increase in the number of iterations. This was because the variation operation should be strong at the incipient iteration to obtain the global optimization ability. On the contrary, the variation operation should be weak at the late iteration to obtain the local development ability. It was not hard to see that the decay coefficient *ξ* was the value of decreasing change between 1 and 0.1, and it controlled the lncRNA components that performed the bitwise inversion in 481 lncRNA components.

### 3.3. Computing *La*

**Figure 5:**
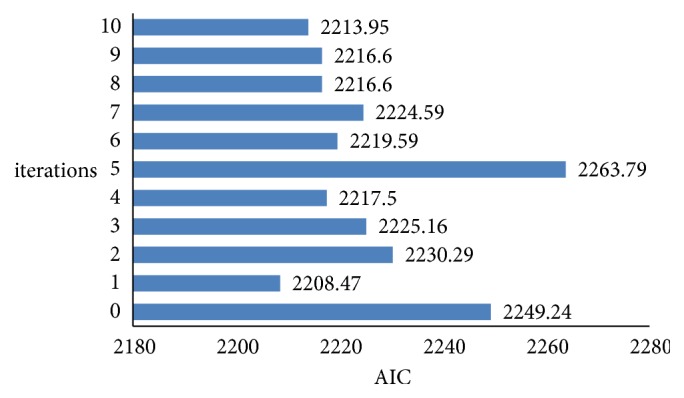
AIC value among 10 iterations.

Table 1 shows the detailed calculation process of *Led*^*sub*^, including the variation position, interval, and result in the *dth* iteration. As shown in Table 1, the variation position and the interval distribution were relatively discrete, reducing the blind area of Γ operation. In addition, the two factors proposed in the calculation to ensure optimal performance were significant differences in *X*_*i*_ (denoted by *SD*_*X*_*i*__) and *AIC* of *Stepwise*(*X*^*d*^) (denoted by *AIC*_*Stepwise*(*X*^*d*^)_), both of which were characterized by the better performance with a smaller value. Two factors should not be considered unilaterally but comprehensively. For example, if only *SD*_*X*_*i*__ was taken into account, *AIC* of *X*_+_^0^, which was the set of *P* less than 10^−12^, was equal to 2249.24, as shown in Figure 5. Obviously, it was not an optimal solution but a poorer solution. Based on the aforementioned considerations, Γ operation, which was proposed in this study, was a combination of *SD*_*X*_*i*__ and *AIC*_*Stepwise*(*X*^*d*^)_. In each variation process, the smaller part of *SD*_*X*_*i*__ performed the variation, rather than the whole. Finally, *X*_+_^1^ (*AIC*=2208.47) was the optimal solution. Therefore, *Led*^*sub*^ was equal to *X*_+_^1^ (i.e., the matrix built using the expression quantity of *X*_+_^1^ on *T*_*i*_, 1 ≤ *i* ≤ 176 ). Then, a multivariate linear regression analysis was performed on *La*, and the regression coefficients were obtained as required (Table 2). The ensemble transcript ID of X14 was ENST00000559477 in the Table 2. The ensemble transcript ID of X16 was ENST00000560882 in the Table 2. The *T* test was performed on the regression model *La* (the results are shown in Table 3). As *P* was less than 0.0001, the regression model *La* had the statistical significance. It also indicated that MlrLDAcp was feasible and effective.

The prediction model (MlrLDAcp) proposed in this study had two potential aspects:

(a) The survival of cancer patients was predicted by combining with the multiple linear regression model of MlrLDAcp.

(b) The association between lncRNAs and diseases was predicted using MlrLDAcp.

The performance of evaluation was expanded from the two aforementioned aspects.

### 3.4. Survival Predictive Ability

**Figure 6:**
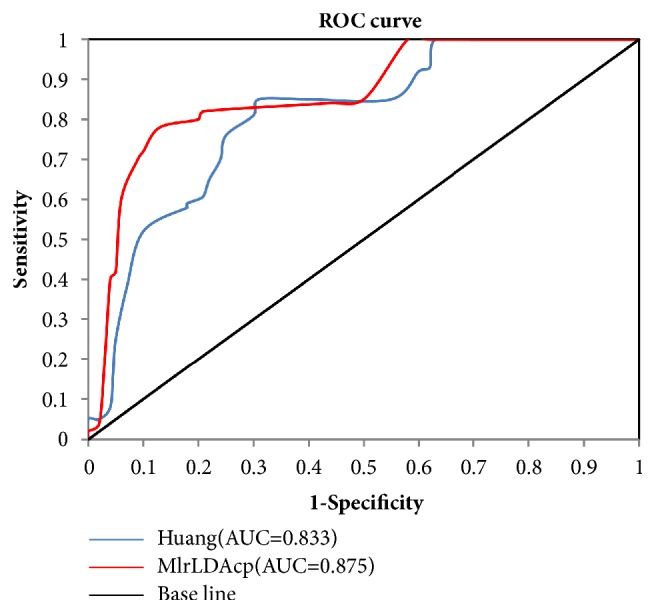
ROC contrast curves of MlrLDAcp and Huang in predicting 5-year biochemical recurrence survival. The prediction accuracy of 5-year biochemical recurrence survival in MlrLDAcp improved by 4.2% (versus Huang).

Receiver operating characteristic (ROC) analyses were performed to compare the predictive accuracies of prostate cancer samples between MlrLDAcp and Huang's method [28] (the state-of-the-art method), to evaluate the survival predictive ability. The 5-year biochemical recurrence survivals of the two methods were compared between TCGA and lncRNAtor databases.  Figure 6 shows the experimental results. The value of the area under the curve (AUC) was calculated from the corresponding area under the ROC curve. As shown in Figure 6, MlrLDAcp with an AUC value of 0.875 was better than Huang with an AUC value of 0.833. As a result, the prediction accuracy of 5-year biochemical recurrence survival in MlrLDAcp was improved by 4.2% (versus Huang). These results suggested that MlrLDAcp might have a predominant survival predictive ability.

### 3.5. Predictive Ability of lncRNA–Disease Associations

**Figure 7:**
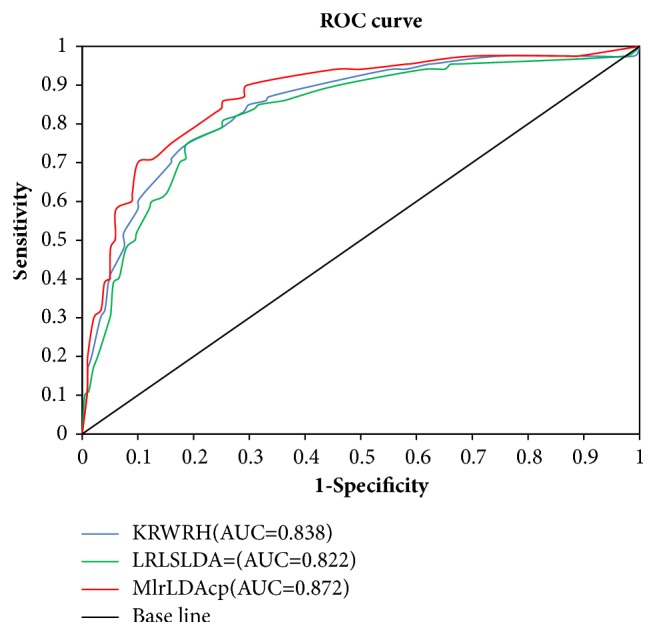
ROC contrast curves of MlrLDAcp and two state-of-the-art methods, LRLSLDA and KRWRH, in predicting lncRNA–disease associations. As can be observed, the prediction accuracy of lncRNA–disease associations in MlrLDAcp improved by 3.4% (versus KRWRH) and 5.0% (versus LRLSLDA).

The leave-one-out cross validation (LOOCV) was implemented on the gold standard dataset to compare MlrLDAcp and two state-of-the-art methods: LRLSLDA [17] and KRWRH [26], to evaluate the predictive ability of lncRNA–disease associations. The datasets were divided into training sets ({*TR*_*S*_}) and test sets ({*TE*_*S*_}). The known lncRNA–disease associations in {*TR*_*S*_} were defined as *K*-*LDA*_*i*_ (1 ≤ *i* ≤ *n*, and *n* was the number of known lncRNA–disease associations). In each step of the LOOCV, each *K*-*LDA*_*i*_ was implemented on {*TR*_*S*_ − *K*-*LDA*_*i*_} and {*TE*_*S*_ + *K*-*LDA*_*i*_}, and then the model learning was carried out on {*TR*_*S*_ − *K*-*LDA*_*i*_}. The ROC curve plotted the sensitivity (that was true-positive rate *TPR* = *TP*/(*TP* + *FN*)) versus the 1-specificity (that was false-positive rate *FPR* = *FP*/(*FP* + *TN*)), where TP denoted true positives, FP denoted false positives, TN denoted true negatives, and FN denoted false negatives. The sensitivity was the ratio of positive samples which could be accurately distinguished, and the specificity represented the percentage of negative samples which could be correctly predicted.  Figure 7 shows the experimental results. The value of AUC was calculated from the corresponding area under the ROC curve. As shown in Figure 7, MlrLDAcp with an AUC value of 0.872 was better than KRWRH with an AUC value of 0.838 and LRLSLDA with an AUC value of 0.822. As a result, the prediction accuracy of lncRNA–disease associations in MlrLDAcp increased by 3.4% (versus KRWRH) and 5.0% (versus LRLSLDA). These results suggested that MlrLDAcp might have a preferable ability to predict lncRNA–disease associations.

## 4. Conclusions

In this study, a model of MlrLDAcp was constructed. MlrLDAcp took the expression quantity of lncRNAs transcript as an independent variable and the clinical prognosis data as a dependent variable. Using MlrLDAcp, 60 lncRNAs, which were most closely related to cancer prognosis information (survival time), were selected from 481 alternative lncRNAs. MlrLDAcp could realize not only the cancer survival prediction but also the lncRNA–disease association prediction.

Further research directions about lncRNA–disease association prediction are as follows.

(a) The lncRNA–disease association prediction should take into account clinical prognostic data in future investigations. The lncRNAs associated with diseases may have a clinical value as therapeutic targets. Hence, the clinical prognostic data is quite valuable to lncRNA–disease association prediction. The clinical implications and the mechanism underlying the association of lncRNAs with diseases are definitely worth exploring further.

(b) How to build an effective computational model to construct an lncRNA similarity function, which can reasonably integrate the similarity scores of different biological information, is worthy of further research.

(c) With the increase in lncRNA–disease correlation, the prediction accuracy can be further improved. Furthermore, most computing models rely heavily on unobtainable negative samples, which is an urgent problem to be solved.

(d) The new network-based computing model should be implemented on heterogeneous networks instead of single networks. Hence, more heterogeneous networks, such as lncRNA–disease network, disease similarity network, lncRNA functional similarity network, and lncRNA interactive networks, should be integrated in the future.

## Figures and Tables

**Table 1:** Variation position, interval, and result of Γ operation in the *dth* iteration.

*d*	*Range*(*X*^*d*^)	*X*_+_^*d*^	LncRNA number in {−*X*_+_^*d*^}
0	----	1–107	{3, 4, 5, 6, 8, 10, 13, 15, 16, 19, 21, 23, 27, 33, 35, 36, 37, 38, 40, 43, 45, 53, 54, 55, 59, 63, 64, 67, 68, 70, 74, 76, 77, 80, 81, 82, 89, 95, 96, 100, 101, 103, 104}
1	(38–441)	(1–37)∪(108–177)	{1, 2, 3, 9, 10, 11, 13, 14, 16, 18, 19, 20, 22, 23, 25, 26, 27, 29, 30, 33, 34, 35, 36, 108, 110, 112, 113, 114, 116, 118, 120, 122, 125, 126, 127, 130, 131, 134, 135, 137, 138, 139, 140, 145, 146, 149, 150, 151, 152, 153, 154, 157, 163, 164, 165, 169, 171, 172, 173, 176}
2	(1–384)	(38–107)∪(178–214)	{40, 41, 43, 44, 45, 46, 47, 50, 70, 76, 87, 89, 90, 98, 99, 101, 104, 182, 185, 186, 192, 194, 198, 199, 200, 203, 207, 214, 59, 202, 74}
3	(106–469)	(108–177)∪(215–251)	{108, 110, 111, 112, 113, 114, 117, 124, 125, 127, 131, 137, 142, 145, 149, 150, 152, 159, 160, 163, 168, 169, 170, 173, 176, 217, 219, 223, 225, 231, 232, 237, 243, 249}
4	(1–312)	(178–214)∪(252–312)	{182, 186, 189, 190, 192, 193, 194, 198, 199, 200, 201, 202, 203, 204, 205, 208, 210, 256, 257, 261, 265, 270, 272, 273, 279, 283, 284, 287, 288, 290, 291, 292, 293, 296, 303, 304, 308, 312}
5	(184–487)	(178–183)∪(215–312)	{178, 182, 265, 270, 272, 273, 274, 277, 288, 289, 292, 293, 301, 302, 305, 306, 307, 312}
6	(1–227)	(184–214)∪(228–303)	{184, 186, 190, 193, 194, 198, 199, 200, 201, 202, 203, 205, 206, 207, 208, 213, 230, 232, 233, 234, 240, 244, 247, 255, 256, 257, 259, 261, 265, 270, 272, 273, 276, 279, 284, 287, 289, 290, 291, 292, 293, 294, 295, 298, 302}
7	(276–481)	(184–214)∪(228–275)∪(304–331)	{186, 190, 194, 197, 200, 201, 202, 203, 205, 208, 214, 228, 229, 230, 233, 240, 241, 243, 245, 247, 257, 261, 265, 268, 270, 272, 273, 306, 309, 310, 317, 319, 327, 329}
8	(1–126)	(108–126)∪(184–214)∪(228–275)∪(304–312)	{108, 110, 112, 114, 121, 124, 125, 126, 184, 190, 193, 194, 197, 198, 200, 203, 206, 208, 210, 213, 214, 228, 230, 232, 233, 235, 238, 239, 241, 247, 248, 249, 254, 256, 257, 262, 265, 267, 268, 270, 272, 273, 306, 308, 309}
9	(389–474)	(108–126)∪(184–214)∪(228–275)∪(304–312)	{108, 110, 112, 114, 121, 124, 125, 126, 184, 190, 193, 194, 197, 198, 200, 203, 206, 208, 210, 213, 214, 228, 230, 232, 233, 235, 238, 239, 241, 247, 248, 249, 254, 256, 257, 262, 265, 267, 268, 270, 272, 273, 306, 308, 309}
10	(1–24)	(1–24)∪(108–126)∪(184–-214)∪(228–260)	{7, 9, 11, 12, 14, 15, 16, 18, 20, 23, 24, 108, 110, 111, 112, 114, 116, 120, 121, 122, 123, 124, 125, 184, 185, 187, 190, 193, 194, 196, 197, 199, 201, 203, 206, 208, 213, 214, 230, 233, 234, 236, 237, 238, 239, 241, 244, 245, 247, 252, 256, 257, 259}

**Table 2:** Results of multiple linear regression analysis on *La* (intercept was constant term, and the rest were 60 independent variables).

Serial number	Gene name	Coefficients	Serial number	Gene name	Coefficients
Intercept	---------	1.486e+03	X120	AMZ2P1	7.286e+07
X1	AC017048.3	–1.808e+08	X122	A2M-AS1	–2.177e+08
X2	KCP	1.669e+09	X125	RP11-399O19.5	–7.673e+07
X3	RP11-342C23.4	–6.523e+07	X126	SNHG16	–7.531e+06
X9	FAM222A-AS1	–9.817e+07	X127	MIR143HG	6.360e+07
X10	PCA3	3.460e+05	X130	GABPB1-AS1	–1.999e+08
X11	CYP4F8	–4.604e+06	X131	GGTA1P	9.237e+07
X13	RP11-627G23.1	–7.704e+07	X134	CTD-2284J15.1	7.787e+07
X14	RP11-279F6.1	–2.877e+07	X135	KB-431C1.4	2.647e+07
X16	RP11-279F6.1	6.888e+07	X137	RP11-66B24.4	–3.950e+07
X18	RP1-163G9.1	2.957e+08	X138	CBR3-AS1	–3.629e+07
X19	AC003090.1	–2.135e+08	X139	MIR22HG	–3.956e+07
X20	AP001626.1	–8.890e+08	X140	DANCR	2.628e+06
X22	AC073133.1	1.038e+08	X145	RRN3P2	4.049e+08
X23	RP11-401F24.4	6.834e+08	X146	LINC00654	–4.514e+08
X25	AC073343.13	6.070e+08	X149	ARHGEF26-AS1	3.073e+07
X26	MAGI2-AS3	–7.389e+07	X150	RMST	–9.826e+07
X27	BOLA3-AS1	1.865e+08	X151	LINC00086	–8.181e+07
X29	C1orf126	–8.830e+08	X152	NBPF8	1.050e+08
X30	CTD-3199J23.4	3.565e+08	X153	CTD-2126E3.1	–1.185e+07
X33	FBXL19-AS1	1.895e+08	X154	AP001258.4	2.169e+07
X34	RPL13P5	–2.848e+08	X157	LINC00312	6.236e+08
X35	RP11-412D9.4	–1.784e+08	X163	RAET1K	–7.308e+08
X36	ADAMTS9-AS2	2.616e+08	X164	PCBP1-AS1	–3.683e+08
X108	XKR5	–1.608e+09	X165	RP11-1000B6.3	3.913e+08
X110	HOXA-AS2	1.159e+08	X169	CTBP1-AS1	3.419e+07
X112	CTC-308K20.1	2.544e+09	X171	BX004987.4	1.583e+08
X113	BX284650.3	1.066e+08	X172	GAS5	–1.064e+07
X114	AC002055.4	–1.601e+08	X173	RP11-166D19.1	–1.299e+08
X116	CD27-AS1	3.876e+07	X176	GBP1P1	–2.108e+08
X118	ATG9B	–1.542e+08			

**Table 3:** * T* test results of multivariate linear regression analysis on *La*.

*T* value test	Residual standard error	*P* value
Value	46.42 on 115 degrees of freedom	1.558e-10

## Data Availability

The data used to support the findings of this study are included within the article.
